# Satisfaction and quality of dying with nonoperative end-of-life care for hospitalized and non-hospitalized frail older patients with (suspected) hip fractures: a combined cohort study

**DOI:** 10.2340/17453674.2025.42998

**Published:** 2025-02-24

**Authors:** Miliaan L ZEELENBERG, Sverre A I LOGGERS, Pieter JOOSSE, Esther M M VAN LIESHOUT, Taco GOSENS, FRAIL-HIP study group, NONU-HIP study group

**Affiliations:** 1 Trauma Research Unit Department of Surgery, Erasmus MC, University Medical Center Rotterdam, Rotterdam; 2 Department of Surgery, Noordwest Ziekenhuisgroep Alkmaar, Alkmaar; 3 Department of Orthopedics, Elisabeth Hospital (ETZ), Tilburg, The Netherlands

## Abstract

**Background and purpose:**

For frail institutionalized hip fracture patients who opt for nonoperative management (NOM), the additional treatment benefits of hospital admission and in-hospital diagnostics are not well evaluated. We aimed to describe and compare treatment satisfaction and quality of dying for patients who refrained from hospitalization after a hip fracture and patients who were treated nonoperatively after a short period of hospitalization.

**Methods:**

Both cohorts included very frail institutionalized hip fracture patients. The first group directly started supportive care in their own nursing home after a suspected hip fracture. The second opted for NOM during shared decision-making after admission and diagnostics at the hospital. Primary outcomes were treatment satisfaction and quality of dying measured by the Quality of Dying and Death Questionnaire (QODD). Secondary outcomes included health-related quality of life (EuroQoL-5D-5L and Qualidem), pain, and medication.

**Results:**

20 non-hospitalized and 88 hospitalized patients were included. Overall treatment satisfaction by proxies was high for both the non-hospitalized 9 (interquartile range [IQR] 8–10) and hospitalized patients 8 (IQR 4–9). Quality of dying was rated higher in the non-hospitalized group with QODD 8.3, IQR 6.9–8.6 versus 7.0, IQR 5.7–7.8, and median difference 1.0 (95% confidence interval [CI] 0.1–1.8). Health-related quality of life, measured by the EQ-5D-5L utility score, was low in both groups but higher in non-hospitalized patients (0.30, IQR 0.15–0.32) than in hospitalized patients (0.25, IQR 0.03–0.32, median difference: 0.03, CI –0.03 to 0.09). Both groups reported similar pain levels, but hospitalized patients used higher standardized daily doses of opiates (68 mg vs 39 mg, median difference 24 mg, CI 7–42).

**Conclusion:**

Proxies of hospitalized and non-hospitalized patients report high treatment satisfaction after opting for NOM. Non-hospitalization may have a beneficial effect on quality of dying in selected patients who have pre-recorded do-not-hospitalize directives or shared decision-making after a suspected hip fracture.

Hip fractures are related with morbidity and mortality in older adults, especially in those with extensive frailty [1,2]. A patient with a hip fracture is associated with limited return to pre-existing mobility and deteriorated health-related quality of life (HRQoL). While effective at reducing pain, operative treatment in frail older adults will increase the risk of surgically associated adverse events and may not always be beneficial to the patient [3,4].

Nonoperative management (NOM) can be an appropriate option for selected frail older patients with poor life expectancy and/or severely limited pre-trauma mobility [5,6]. In patients favoring palliative care focused on HRQoL and comfort, NOM may even be a more appropriate approach than operative management focused on return to function [7,8]. Currently, NOM is an increasingly used treatment option in the Netherlands, at around 3–5% of the total annual number of hip fractures in 2022 [9,10].

In frail older patients with hip fractures, non-inferior HRQoL for NOM with high treatment satisfaction and quality of dying has been reported [11]. In a case series of nursing home patients with suspected hip fractures with do-not-hospitalize directives or advance care plans, high treatment satisfaction and quality of dying, reported by proxies and caregivers, was found [12].

Not hospitalizing patients (due to advance care plans or do-not-hospitalize directives) after a suspected hip fracture could potentially spare them from needless transfers, testing, and complications associated with hospital admissions, while also allowing them to directly receive care in a trusted environment. More comparative information can aid physicians and surgeons during advance care planning or decision-making after suspected hip fractures and help them provide patients with more detailed expectations.

Therefore, we aimed to describe and compare treatment satisfaction and quality of dying in 2 cohorts of frail institutionalized patients with a (suspected) hip fracture, 1 group that was presented to the emergency department for diagnostics and decision-making or underwent a short period of hospitalization and 1 that was not hospitalized due to prerecorded do-not-hospitalize directives or pre-hospital decision-making.

## Methods

### Study design

A retrospective cohort study was conducted using data collected by the NONU-HIP [12] and FRAIL-HIP [11] studies. These studies were conducted, respectively, from November 1, 2019 to December 31, 2022 and from September 1, 2018 to April 25, 2020. They are reported using the STROBE guidelines.

### Population

This study described and compared 2 groups of patients, a non-hospitalized group and a hospitalized group. The non-hospitalized group consisted of patients included in the NONU-HIP study. This was a prospective case series in 3 nursing homes in the Netherlands. All permanently institutionalized nursing home patients aged over 65 years with a suspected hip fracture, as judged by the elderly care physicians or nurse practitioner, who underwent NOM without hospital admission were included. Treatment decisions for NOM were either made through shared decision-making with patients and/or legal representatives with medical professionals in the hospital and for non-hospitalized patients, shared decision-making in their institution of residence or because of pre-existing do-not-hospitalize directives from advance care plans.

The hospitalized group included patients from the FRAIL-HIP study. This was a 6-month prospective cohort study at 25 hospitals across the Netherlands. Eligible patients were aged 70 years or older, frail, institutionalized, and sustained a femoral neck or trochanteric fracture. The term frail implied that at least 1 of the following characteristics was present: malnutrition (body mass index < 18.5) or cachexia, severe comorbidities (American Society of Anesthesiologists physical status class of IV or V), or mobility issues (Functional Ambulation Category ≤ 2). Only data from patients who chose NOM was included in this analysis. These patients either died during their hospital stay or returned to an institution or hospice after opting for NOM.

### Outcome measures and data collection

The primary outcome measures were treatment satisfaction and quality of dying, using the Quality of Dying and Death (QODD) questionnaire [13], both reported by proxies. Secondary outcome measures were HRQoL using by the EuroQol 5-Dimension 5-Level (EQ-5D-5L) questionnaire and Qualidem questionnaire [14], pain assessed by the Pain Assessment Checklist for Seniors With Limited Ability to Communicate (PACSLAC) [15], and daily amount of narcotic drug administration (total used dose during follow-up) was converted into equivalent doses of 1 mg of oral morphine per day. Additional data collected was: age, sex, BMI, KATZ-ADL score, cognitive function, sedative medication use, and mortality.

Treatment satisfaction, reported by proxies, was measured on a numeric rating scale (0–10) after a patient had died or at the end of the 6-month follow-up. Herein, 0 was extremely dissatisfied and 10 extremely satisfied. Quality of death and dying was also assessed by proxies, after patients’ deaths, using the QODD questionnaire [13]. Currently no minimal clinically important difference (MCID) has been calculated for the QODD.

This interview-based questionnaire explores 17 end-of-life priorities resulting in 3 corresponding categories of the quality of the final period of the decedent’s life (“terrible–poor,” “intermediate,” and “good–almost perfect”). It also distinguishes 4 subdomains: symptom control, (death) preparation, connectedness (i.e., sharing physical expressions of affection, spending time with family/friends), and transcendence (i.e., being unafraid of and at peace with dying). The EQ-5D-5L, also reported by proxies, is an instrument for measuring HRQoL, consisting of a utility score (EQ-US) and a visual analog scale (EQ-VAS) [16]. It was converted into utility scores using the Dutch tariff [17]. The Qualidem, a 37-item questionnaire completed by caregivers covering the last 7 days, measured the HRQoL on 9 domains in persons with dementia [14]. The items are rated on a 4-point Likert scale. Higher scores indicate better HRQoL (minimum score 0, maximum scores between 6 and 21 per domain). The PACSLAC is a 24-item instrument for recognizing pain in patients with dementia and was completed by caregivers [15]. The total score ranges from 0 to 24, resulting in a dichotomous outcome with a score of > 4 points indicating significant pain. Because of the degree of cognitive impairment of both study groups, questionnaires completed by proxies and/or caregivers were inevitable

In this analysis, measurements on HRQoL and pain were compared for the follow-up moment within the 1st week posttrauma, around 3 days for the non-hospitalized group (NONU-HIP study) and 7 days for the hospitalized group (FRAIL-HIP study). As almost all patients in the non-hospitalized, and a large proportion of hospitalized patients died before or shortly after 7 days, follow-up calculation for other time-points was not possible.

### Statistics

Descriptive analysis was performed using SPSS version 25.0 (IBM Corp, Armonk, NY, USA). Continuous data was reported as median and interquartile ranges (IQR) (due to non-normal distributions), categorical data as number with percentages. For categorical data absolute differences with 95% confidence interval (CI) were calculated using MedCalc Statistical Software, version 18.2.1 (MedCalc-Software Ltd, Ostend, Belgium). For continuous variables, the Hodges–Lehmann estimator was used to calculate median differences with corresponding 95% CIs.

### Ethics, data sharing, funding, use of AI, and disclosures

Both the FRAIL-HIP study (ref.no 2018.208) and NONU hip study (ref.no 2019.343) were exempted by the Medical Research Ethics Committee of VUmc. Patients or proxies provided written consent for participation. No funding was received for this analysis. The data that support the findings of this study is available from the corresponding author upon reasonable request. AI was not used in the analysis or writing of this manuscript. The authors declare no conflicts of interest. Complete disclosure of interest forms according to ICMJE are available on the article page, doi: 10.2340/17453674.2025.42998

## Results

**Figure 1:**
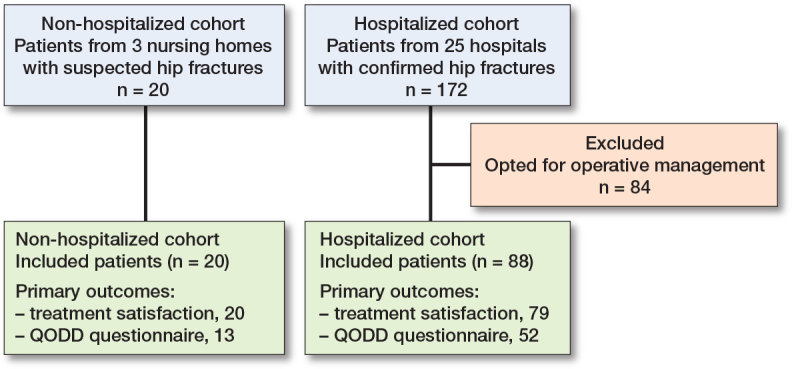
Flowchart of patients for both included cohorts and the number of patients with data available for the primary outcomes. QODD = Quality of Dying and Death questionnaire.

108 patients were included in the analysis, 20 patients in the non-hospitalized group and 88 in the hospitalized group (Figure 1). The total population had a median age of 88 years (IQR 84–93) and 83 (77%) patients were female (Table 1). Both groups were similar in age, sex, BMI, and ASA score. None of the patients functioned without assistance in any measured activities of daily life, but a higher proportion of hospitalized patients had a KATZ of > 4. Of hospitalized patients, 49 (55%) were admitted to the hospital for at least 1 day, with a median stay of 2 days (IQR 2–3). The others returned to their institution of residence after the diagnostic process and shared decision-making at the emergency department. For non-hospitalized patients a pre-defined do-not-hospitalize directive was in place in 9 patients, and in the other 11 cases NOM was started after shared decision-making with the patient and/or next-of-kin. 30-day mortality was 100% in non-hospitalized patients and 83% in hospitalized patients (absolute difference 17%, CI –0.3 to 27). Additional injury and treatment characteristics showed a median hospital stay of 2 days (IQR 2–3) for hospitalized patients. Among hospitalized patients, 54 (61%) suffered a femoral neck fracture and 30 (39%) a trochanteric hip fracture (Table 2, see Supplementary data).

**Table 1:** Baseline characteristics. Data is shown as median (IQR) or as n (%)

Characteristic	Non-hospitalized		Hospitalized		Absolute (in %) or median ^a^ difference (CI)
	n ^b^	(n = 20)	n ^b^	(n = 88)	
Age	20	87 (82–92)	88	89 (84–93)	–1 (–4 to 2)
Female sex	20	16 (80)	88	67 (76)	4 (–18 to 19)
BMI, median	12	21 (19–22)	68	21 (18–26)	0 (–2 to 3)
Nursing home resident	20	20 (100)	88	88 (100)	0 (–4 to 16)
Hospital admission after					
hip fracture diagnosis	20	NA	88	49 (56)	NA
Dementia diagnosis	20	17 (85)	88	83 (94)	9 (–3 to 30)
Katz ADL score	20				
0		0 (0)	84	0 (0)	0 (–16 to 4)
1		2 (10)		0 (0)	10 (2 to 30)
2		1 (5)		4 (5)	0 (–18 to 8)
3		2 (10)		13 (16)	6 (–1 to 18)
4		7 (35)		12 (14)	21 (2 to 43)
5		5 (25)		23 (27)	2 (–21 to 19)
6		3 (15)		32 (38)	23 (–0.1 to 37)
ASA category	20				
II		2 (10)	88	4 (5)	5 (–4 to 25)
III		15 (75)		63 (60)	15 (–9 to 32)
IV		3 (15)		31 (35)	20 (–3 to 34)
Days to death, median	20	5 (3–6)	83	7 (5–12)	–3 (–5 to –1)
30-day mortality	20	20 (100)	88	73 (83)	17 (–0.3 to 26)

ADL = activities of daily living; BMI = body mass index; CCI = Charlton Comorbidity Index; FAC = Functional Ambulation Category; IQR = interquartile range.

a Median differences (and 95% confidence intervals) were calculated using the Hodges–Lehmann estimator.

b Number of patients for whom data was available.

**Figure 2:**
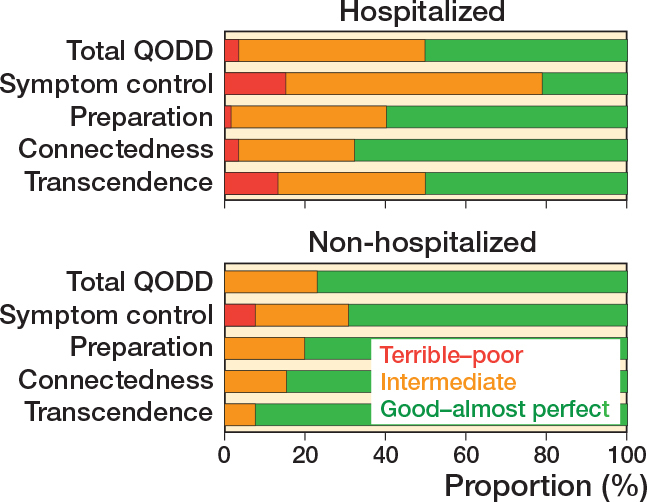
Quality of dying rated by proxies measured by the QODD questionnaire for non-hospitalized and hospitalized patients with ratings for the combined (total) QODD and the 4 subdomains.

Overall treatment satisfaction, measured by proxies, was high for both groups, with a median NRS of 9 (IQR 8–10) in non-hospitalized patients and 8 (IQR 8–10) for hospitalized patients with a median difference of 1 (CI 0–1) (Table 3). Quality of dying, measured by the QODD total score, was rated better (median difference 1.0, CI 0.1–1.8) by proxies of non-hospitalized patients (8.3, IQR 6.9–8.6) than in hospitalized patients (7.0, IQR 5.7–7.8). 10 proxies (76%) of the non-hospitalized group and 26 proxies (50%) of the hospitalized group rated the quality of dying as “good–almost perfect” (Figure 2). The subscores for the domains symptom control, preparation, and transcendence were also rated higher in the non-hospitalized group. Symptom control was the worst rated subcategory for both groups, with more frequent “good–almost perfect” ratings for non-hospitalized patients across all 4 domains (Figure 2). For an overview of all questions per subcategory (Figure 3, see Supplementary data).

**Table 3:** Primary (treatment satisfaction and quality of dying) and secondary outcomes (health-related quality of life, pain and medication) for non-hospitalized and hospitalized patients. Data is shown as median (IQR) or as n (%)

Characteristic	Non-hospitalized		Hospitalized		Absolute (in %) or median ^a^ difference (CI)
	n ^b^	(n = 20)	n ^b^	(n = 88)	
**Primary**					
Treatment satisfaction					
NRS proxy	20	9 (8–10)	79	8 (4–9)	1 (0 to 1)
QODD					
Total score	13	8.3 (6.9–8.6)	52	7.0 (5.7–7.8)	1.0 (0.1 to 1.8)
Symptom control	13	7.7 (6.7–8.3)	52	5.7 (3.1–6.7)	2.0 (1.0 to 3.3)
Preparation	15	8.8 (8.0–9.2)	52	7.0 (6.0–8.0)	1.4 (0.6 to 2.1)
Connectedness	15	8.5 (7.8–9.0)	52	7.5 (6.0–9.0)	1.0 (0.0 to 1.5)
Transcendence	15	8.7 (7.7–9.0)	52	6.8 (4.8–8.0)	1.7 (0.7 to 2.7)
**Secondary**					
EQ-5D-5L					
Utility proxy	18	0.30 (0.15–0.32)	83	0.25 (0.03–0.32)	0.03 (–0.03 to 0.09)
VAS proxy	18	25 (18–46)	83	40 (30–50)	–10 (–20 to 0)
Qualidem **^c^**					
Care relationships (0–21)	14	20 (18–21)	84	19 (17–21)	1 (0 to 2)
Positive affect (0–18)	14	10 (6–11)	84	9 (5–12)	1 (–1 to 4)
Negative affect (0–9)	14	9 (7–9)	85	7 (5–9)	1 (0 to 2)
Restless tense behavior (0–9)	14	5 (3–7)	85	4 (2–6)	0 (–1 to 2)
Positive self-image (0–9)	14	9 (8–9)	85	9 (9–9)	0 (0 to 0)
Social relations (0–18)	14	8 (6–10)	83	7 (5–9)	1 (–1 to 3)
Social isolation (0–9)	14	6 (4–6)	85	6 (6–8)	0 (–1 to 0)
Feeling at home (0–12)	14	12 (11–12)	84	12 (12–12)	0 (0 to 0)
Having something to do (0–6)	14	0 (0–1)	84	0 (0–0)	0 (0 to 0)
Pain and medication **^c^**					
PACSLAC **^c^**					
during care (0–24)	19	5 (4–10)	85	6 (4–9)	0 (–2 to 2)
> 4 during care, n (%)	19	17 (90)	85	75 (88)	2 (–19 to 13)
Daily morphine					
administration **^d^**	20	39 (16–65)	88	68 (39–95)	–24 (–42 to –7)
Palliative sedation, n (%)	20	14 (70)	81	41 (50)	20 (–4 to 39)

IQR = interquartile range; NRS = numeric rating scale; QODD = Quality of Dying and Death questionnaire; VAS = visual analog scale. PACSLAC = Pain assessed by the Pain Assessment Checklist for Seniors With Limited Ability to Communicate.

a Median differences (and 95% confidence intervals) were calculated using the Hodges–Lehmann estimator.

b Number of patients for whom data was available.

c Both Qualidem and PACSLAC scores were completed by caregivers instead of proxies.

d Total morphine administration was calculated by conversion of all administered opioid pain medication into mg of oral morphine per day for the first week after trauma.

**Figure 4:**
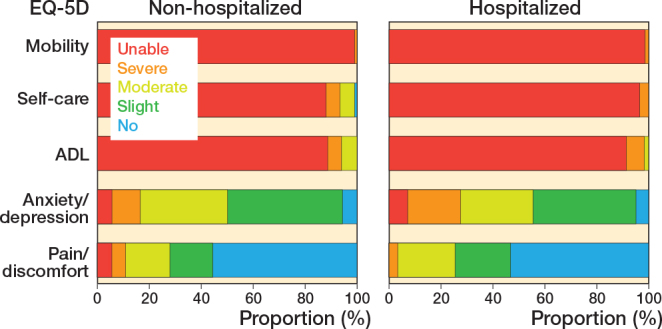
Quality of life for non-hospitalized versus hospitalized patients measured by the EQ-5D-5L for the 5 subdomains by proxies during the first week after trauma.

HRQoL measured by the EQ-5D-5L through proxies was low for both groups (Table 3). While the VAS scores were similar, the utility score was higher in non-hospitalized patients (0.30, IQR 0.15–0.32) than in hospitalized patients (0.25, IQR 0.03–0.32, median difference: 0.03, CI –0.03 to 0.09). However, the magnitude of this difference does not seem clinically relevant, based on nonspecific MCID values [18]. Proxies reported high disability with respect to mobility, self-care, and ADL tasks for both non-hospitalized and hospitalized patients (Figure 4). Extreme pain and anxiety/depression was uncommon in both groups. HRQoL measured by the domains of the Qualidem score registered high (positive) scores for both groups in the categories “care relationships,” “feeling at home,” and “positive self-image,” and lower (negative) scores in the categories “having something to do,” “social relations,” and “restless tense behavior.”

Pain during care moments, measured using the PACSLAC, was similar between the groups, with a median score of 5 (IQR 4–10) for non-hospitalized and 6 (IQR 4–9) for hospitalized patients (median difference 0, CI –2 to 2) (Table 3). Non-hospitalized patients received a lower median dose of morphine per day of follow-up, 39 mg (IQR 16–65) versus 68 mg (IQR 39–95) (median difference –24 mg, CI –42 to –7). In 17 (70%) of non-hospitalized and 41 (51%) of hospitalized patients, palliative sedation was started during the final phase of life (absolute difference 20%; CI –4 to 39).

## Discussion

We aimed to describe and compare treatment satisfaction and quality of dying for patients who refrained from hospitalization after a (suspected) hip fracture and patients who were treated nonoperatively after a short period of hospitalization. We found that proxies of both hospitalized and non-hospitalized patients who opt for NOM report high treatment satisfaction and high quality of dying for the majority of patients. Additionally, non-hospitalization may have beneficial effects on quality of life and dying in a select group of very frail older adults who opt for non-hospitalization after a pre-recorded do-not-hospitalize directive or shared decision-making after a suspected hip fracture.

This is the first study describing and comparing data from hospitalized and non-hospitalized patients after opting for NOM for a (suspected) hip fracture. These outcomes can aid physicians in shared decision-making and advance care planning, and help them provide more realistic expectation management, or serve as a starting point for further study. However, due to the selection of both populations, the different moments at which patients opted for NOM, and differences in pre-existing expectations or preparedness of death or dying there is a high risk of selection bias, almost by definition. Interpretation and extrapolation of results should therefore be done with considerable care.

Proxies in both groups reported high treatment satisfaction and quality of dying, comparable to results found in operatively managed patients [11]. However, proxies of non-hospitalized patients reported better scores in the QODD total score and the subcategories symptom control, preparation, and transcendence. This can be interpreted as less observed pain, a sense of less strain on loved ones, and patients seeming more at peace with dying. The minimal important change for the QODD is unknown, making the clinical value of these differences, of a median 1–2 points per subcategory, unclear. Non-hospitalized patients did show a higher proportion of patients in “good–excellent quality of dying” and no proxies who rated the quality of dying below intermediate, suggesting that in this sample there were advantages for the non-hospitalized group. Previous qualitative studies reported that some of the most important aspects in comfortable palliative care after hip fracture are control of pain, easy access to and communication with loved ones, and a calm and peaceful moment of dying [19]. Other studies also identified maintaining quality of life (over prolonging of life) and being with family as more important goals of care than hospital admission and intensive treatment for frail patients with dementia [20,21]. Transfers to and within the hospital, diagnostics, and care moments in an unfamiliar environment may all have contributed to the lower quality of dying of those who were hospitalized, as these will have placed more strain on both patients and next-of-kin, and potentially increase the risk of delirium. Alternatively, non-hospitalized patients and their next-of-kin may have been more at peace with dying or prepared for the consequences of NOM due to predetermined advance care plans or do-not-hospitalize directives. Dying within a short timespan would have been within the realm of expectation.

Pain during care was rated similar for both groups and is often one of the most challenging areas of NOM. Despite similar pain scores and a small proportion of patients receiving a local nerve block, hospitalized patients used higher mean doses of opiates per day. This might be due to differences in protocols, in availability of medication, and means of administration between hospitals and nursing homes. It might also indicate that hospitalized patients experienced higher pain levels, therefore needing more analgesia. Conversely, some non-hospitalized patients may have benefited from additional pain medication to further decrease pain levels. A larger proportion of the non-hospitalized started palliative sedation, which may have reduced the need for or use of additional pain medication.

HRQoL during the first week after trauma was rated low in both groups by proxies or caregivers, with the small difference falling below most nonspecific MICD values [18]. This can be expected, as both groups included very frail patients in their final phase of life, often with pre-existing low HRQoL due to age, comorbidities, or mobility issues, worsened by the hip fracture [2,19]. The process of hospitalization seems not to have worsened these scores, although several patients in both groups scored near the absolute minimum of the EQ-5D-5L and several of the Qualidem subcategories.

This study shows a notably longer survival in the hospitalized group, which most likely is explained by selection bias. The main reason for this difference might be that many in the non-hospitalized group had do-not-hospitalize directives based on other diagnoses or end-of-life preferences and consequently were treated earlier in a palliative manner. The longer survival combined with low HRQoL may also have negatively impacted quality of dying in this group, as judged by proxies, due to longer periods of ongoing perceived suffering [19]. However, others may have valued the additional time with their loved ones. Is unlikely that the process of hospitalization will have significantly prolonged survival. For a large proportion of the hospitalized patients the decision for nonoperative palliative care had to be made after trauma in an acute hospital setting. This could possibly have led to a broader range of patients opting for NOM, with less specific advance care plans and wishes for end-of-life care and a less predictable short-term mortality.

When interpreting this study’s results, basic differences between countries and their healthcare systems must be considered. NOM is starting to gain more international attention and acceptance but is still a small area of research [22,23]. Current projects in the Netherlands focus on implementation of standardized shared decision making between operative management and NOM in the hospital setting for selected frail elderly with hip fractures [24]. However, international views, culture, and legislation on hip fracture care in the final phase of life differ greatly and not all will consider NOM a favorable alternative to surgery [23].

As it is unclear who out of all frail institutionalized patients benefits from either operative management or NOM, both approaches need to be discussed.

### Limitations

We suspected selection bias as the major limitation. The sample size is small and the smaller group size of the non-hospitalized group may have affected the generalizability of results. The few data points for several outcomes hamper the possibility of matching or viability of regression analysis. However, both groups were similar in terms of age, sex, comorbidities, pre-trauma living situation, and mobility. While conducted within the first week for both groups, follow-up did not occur on the same day after fracture in the groups. Due to the nature of these patients, with high rates of dementia and/or sedative medication, the included studies were able to assess only HRQoL and quality of dying through proxies or caregivers.

### Conclusion

Proxies of hospitalized and non-hospitalized patients who opt for NOM reported high treatment satisfaction and high quality of dying for a majority of patients. In the situation of a suspected hip fracture, opting for non-hospitalization after a prerecorded do-not-hospitalize directive or shared decision-making may have benefits for quality of dying in selected very frail older adults when compared with patients who were hospitalized for diagnostics and in-hospital shared decision-making.

*In perspective,* future efforts should focus on studying and improving shared decision-making and advance care planning in frail institutionalized older adults with a hip fracture so that hospitalization can be prevented for those patients who would prefer familiar care focused on comfort and quality of life in their final moments.

Refraining from hospitalization for selected patients who prefer nonoperative end-of-life care decreases the amount of unneeded in-hospital diagnostics and interventions, which results in a cost decrease for the health are system of approximately €2,200 per patient [25,26]. Therefore, it is important to improve identification of patients for whom hospitalization, diagnostics, and/or advanced pain treatment would be either beneficial or redundant after a suspected hip fracture. However, the cornerstone of both patient satisfaction and quality of dying remains the thorough and timely discussion of expectations and wishes for the final phase of life, ideally conducted before a fracture occurs.

### Supplementary data

Table 2 and Figure 3 are available as supplementary data on the article page, doi: 10.2340/17453674.2025.42998

## Supplementary Material



## Notes

Conceptualization and design of the study: SL, PJ, and EvL. Supervision: EvL and TG. Data collection: SL. Data-analysis: MZ, SL, and EvL. Drafting of the manuscript: MZ. Critical revision and approval of the manuscript for submission: MZ, SL, PJ, EvL, and TG.

Handling co-editors: Cecilia Rogmark and Robin Christensen

*Acta* thanks Antony Johansen and Bjarke Viberg for help with peer review of this manuscript.
