# Supplementary material for: Satisfaction and quality of dying with nonoperative end-of-life care for hospitalized and non-hospitalized frail older patients with (suspected) hip fractures: a combined cohort study
Source: Acta Orthop. 2025 Feb 24;96:167–73. doi: 10.2340/17453674.2025.42998 (PMC11849162; doi:10.2340/17453674.2025.42998)
Supplement: Supplementary file 1 [file ActaO-96-42998-s1.pdf]

Supplemental tables and figures

Table 2. Injury characteristics for hospitalized patient and additional treatment characteristics for both groups

	**Non-hospitalized (n = 20)**		**Hospitalized (n = 88)**	
**Characteristic**	n [a]	n	n [a]	median (IQR) or n (%)
Fracture type				
Femoral neck	Unknown	Unknown	54	54 (61)
Pertrochanteric	Unknown	Unknown	34	34 (39)
Days of hospital stay (IQR)	NA	NA	49	2 (2–3)
Consultations [b]				
Physical therapist	20	5	88	39 (44)
Occupational therapist	20	2	88	31 (35)
Dietician	20	1	88	8 (9.1)
Spiritual counselor	20	1	88	2 (2.3)
Antipsychotic medication [b]	20	2	88	29 (33)
Femoral nerve block	20	NA	88	14 (16)
PENG block	20	NA	88	1 (1.1)
Registered adverse events [b]	20	2	88	35 (40)
Pressure ulcers	20	0	88	20 (20)
Delirium	20	2	88	8 (9.1)
Heart failure	20	0	88	2 (2.3)
Urinary tract infection	20	0	88	4 (4.5)
Pneumonia	20	0	88	2 (2.3)
Fall	20	0	88	1 (1.1)
Morphine intoxication	20	0	88	1 (1.1)

[a] number of patients for whom data were available.

[b] Up to 1 week follow-up

ED = emergency department; NA = not applicable; PENG = pericapsular nerve group**;** IQR = interquartile range

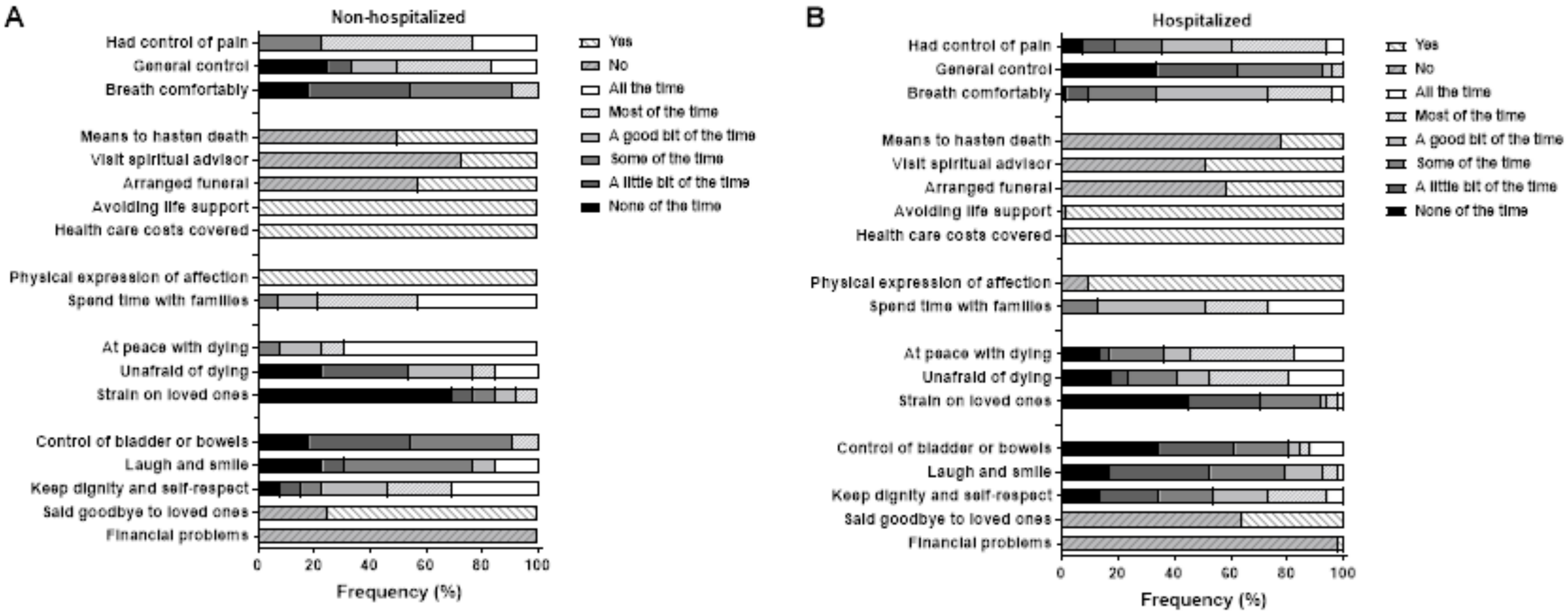

Figure 3. Comparison of individual QODD questions for non-hospitalized (A) and hospitalized (B) patients
